# Is CAA a perivascular brain clearance disease? A discussion of the evidence to date and outlook for future studies

**DOI:** 10.1007/s00018-024-05277-1

**Published:** 2024-05-27

**Authors:** Susanne J. van Veluw, Helene Benveniste, Erik N. T. P. Bakker, Roxana O. Carare, Steven M. Greenberg, Jeffrey J. Iliff, Sylvie Lorthois, William E. Van Nostrand, Gabor C. Petzold, Andy Y. Shih, Matthias J. P. van Osch

**Affiliations:** 1 Department of Neurology, Massachusetts General Hospital, Harvard Medical School, Boston, MA USA; 2 Department of Anesthesiology, Yale School of Medicine, New Haven, CT USA; 3 Department of Biomedical Engineering, Amsterdam University Medical Center, Location AMC, Amsterdam Neuroscience Research Institute, Amsterdam, The Netherlands; 4 Clinical Neurosciences, University of Southampton, Southampton, UK; 5 VA Puget Sound Health Care System, University of Washington, Seattle, WA USA; 6 Institut de Mécanique Des Fluides de Toulouse, IMFT, Université de Toulouse, CNRS, Toulouse, France; 7 Department of Biomedical and Pharmaceutical Science, George & Anne Ryan Institute for Neuroscience, University of Rhode Island, Kingston, RI USA; 8 German Center for Neurodegenerative Disease, Bonn, Germany; 9 Division of Vascular Neurology, Department of Neurology, University Hospital Bonn, Bonn, Germany; 10 Center for Developmental Biology and Regenerative Medicine, Seattle Children’s Research Institute, University of Washington, Seattle, WA USA; 11 Department of Radiology, Leiden University Medical Center, Leiden, the Netherlands

**Keywords:** Glymphatics, IPAD, Perivascular spaces, Cerebral amyloid angiopathy, Cerebrospinal fluid, Brain clearance

## Abstract

The brain’s network of perivascular channels for clearance of excess fluids and waste plays a critical role in the pathogenesis of several neurodegenerative diseases including cerebral amyloid angiopathy (CAA). CAA is the main cause of hemorrhagic stroke in the elderly, the most common vascular comorbidity in Alzheimer’s disease and also implicated in adverse events related to anti-amyloid immunotherapy. Remarkably, the mechanisms governing perivascular clearance of soluble amyloid β—a key culprit in CAA—from the brain to draining lymphatics and systemic circulation remains poorly understood. This knowledge gap is critically important to bridge for understanding the pathophysiology of CAA and accelerate development of targeted therapeutics. The authors of this review recently converged their diverse expertise in the field of perivascular physiology to specifically address this problem within the framework of a Leducq Foundation Transatlantic Network of Excellence on Brain Clearance. This review discusses the overarching goal of the consortium and explores the evidence supporting or refuting the role of impaired perivascular clearance in the pathophysiology of CAA with a focus on translating observations from rodents to humans. We also discuss the anatomical features of perivascular channels as well as the biophysical characteristics of fluid and solute transport.

## Outline and scope of the review

Maintenance of cerebral homeostasis and function not only requires constant supply of vital nutrients and oxygen to the brain, but also continuous drainage of metabolic waste products. Our knowledge of brain clearance has been surprisingly limited until recent discoveries demonstrated that perivascular compartments along the vasculature are critical for facilitating drainage from the brain [1–3]. Impaired perivascular brain clearance of soluble amyloid β (Aβ) has been implicated in the pathophysiology of cerebral amyloid angiopathy (CAA), where Aβ accumulates in the walls of capillaries and arterioles, and occasionally venules [4, 5]. The exact mechanisms through which perivascular brain clearance and vascular Aβ depositions are linked, however, remain poorly understood. This knowledge gap is critical, since CAA is the main cause of hemorrhagic stroke, an important contributor to cognitive decline in older individuals, and the most common vascular comorbidity in Alzheimer’s disease (AD) [6, 7]. More recently, CAA has been recognized as a condition that might explain the occurrence of adverse events in the form of amyloid-related imaging abnormalities (ARIA) in AD patients receiving anti-amyloid immunotherapy [8–10]. With the aging population, CAA is increasing, while effective disease-modifying interventions are nonexistent [11]. Unraveling the mechanisms of perivascular brain clearance will have major implications for understanding CAA as well as other common dementia disorders. Moreover, understanding how brain clearance can potentially be enhanced, for example by improving sleep or other key physiological drivers, is crucial not only for CAA patients but for the aging population at large.

Two key overarching and fundamental knowledge gaps have hampered our understanding of the role of impaired perivascular brain clearance in CAA and thereby also the possible development of treatment strategies aimed at improving Aβ clearance: (1) Significant controversies remain in the field regarding the anatomical pathways, directionality, and mechanistic driving forces of brain clearance, and how they are altered during the early and late stages of CAA. These discrepancies have in part arisen from a lack of detailed characterization of the microanatomical structures implicated in clearance both in the rodent and human brain, heterogeneous experimental imaging approaches, the use of different rodent models and anesthetic regimens, artifacts related to focal tracer injections, and post-mortem fixation. This has resulted in discrepant findings and data interpretation across labs, which have also slowed translational impact. (2) It is unclear whether observations in rodent models can be readily translated to the human brain. A major obstacle to understanding brain clearance in humans has been the lack of non-invasive imaging techniques for perivascular brain clearance. The development and validation of novel MRI sequences, capable of measuring interstitial fluid (ISF) and cerebrospinal fluid (CSF) mobility and water exchange between compartments as surrogates for brain clearance, might accelerate translational efforts and allow studying perivascular brain clearance in healthy individuals and CAA patients.

The goal of this review paper is to critically assess the evidence supporting or refuting the role of impaired perivascular brain clearance in the pathophysiology of CAA. Moreover, we aim to provide an outlook for future studies needed to answer existing fundamental unknowns. The authors are members of the Leducq Foundation Transatlantic Network of Excellence on Brain Clearance [12]*.* The key motivation that brought this consortium together is that the fundamental brain clearance knowledge gaps and controversies cannot be bridged by any individual or lab alone. Rather, it requires collaboration as well as debate between pre-clinical, clinical, and computational investigators, who approach brain clearance from different viewpoints, using complementary techniques. The goal of its members with complementary technical expertise and viewpoints is to resolve conflicting experimental findings and to translate observations made in rodents to the human brain, thereby maximizing translational impact. As such, the overarching consortium aims are to (1) establish a physiologically and pathologically informed, integrated multi-scale understanding of perivascular brain clearance in health and CAA, (2) translate experimental findings from rodents to the human brain, and (3) identify relevant driving forces to be tested in future clinical trials to enhance soluble waste clearance for brain health.

## Introduction to cerebral amyloid angiopathy (CAA)

### Demographics

CAA is a common age-related pathology and a major cause of spontaneous lobar intracerebral hemorrhage (ICH) as well as a significant contributor to age-related cognitive decline and disability. Systematic review and meta-analysis of autopsy studies performed in unselected older individuals representative of the general population reported 41.5% (95% confidence interval [CI] 33.1–50.2%) prevalence of mild-to-severe CAA and 23.0% (95% CI 17.3–29.1%) prevalence of moderate-to-severe CAA [7]. These figures greatly exceed the incidence of lobar ICH [13, 14] the most salient clinical presentation of CAA [5], indicating that CAA pathology is most often clinically undetected. Even in the absence of acute presentations such as ICH, however, moderate-to-severe CAA is independently associated with impaired cognitive performance during life [15], placing it among the common vascular contributors to cognitive impairment and dementia [16].

CAA occurs at increased prevalence in the presence of AD pathology (i.e. in the presence of parenchymal Aβ plaques and intracellular tau tangles), presumably reflecting the central role of Aβ accumulation in both disorders [4]. Meta-analysis of autopsy studies performed in individuals with AD found mild-to-severe CAA in 79.2% (95% CI 72.5–85.3%) and moderate-to-severe CAA in 47.5% (95% CI 38.8–56.2%) [7]. Other than age, the best characterized risk factor shared by CAA and AD is presence and number of apolipoprotein E (*APOE*) ε4 alleles, which has been associated with both greater AD and CAA severity [17]. Other factors associated with Aβ deposition also promote both CAA and AD pathology, but often one in preference to the other. For example, mutations of the Amyloid Precursor Protein (*APP*) gene that increase the ratio of the longer Aβ_42_ to the shorter Aβ_40_ species (such as the London Val717Ile mutation), cause primarily AD pathology, whereas several *APP* missense mutations within the Aβ-coding sequence (such as the Dutch Glu693Gln mutation) strongly favor CAA [4]. Of note, severe CAA is a common feature of *APP* mutations within the Aβ-coding sequence, but clinical manifestations (ICH, cognitive impairment, or both) differ, sometimes even between individuals carrying the same mutation [18]. Other genetic causes of AD with CAA include *PSEN1* and *PSEN2* mutations and copy number variants of *APP* (including trisomy 21). There is a further class of CAA risk factors that potentiate steps in CAA pathogenesis downstream from initial vascular Aβ deposition [5]. The most prominent of these is the *APOE* ε2 allele, which is partially protective from AD pathology but increases risk of CAA-related vessel wall breakdown and ICH [19].

### Neuropathology

**Fig. 1:**
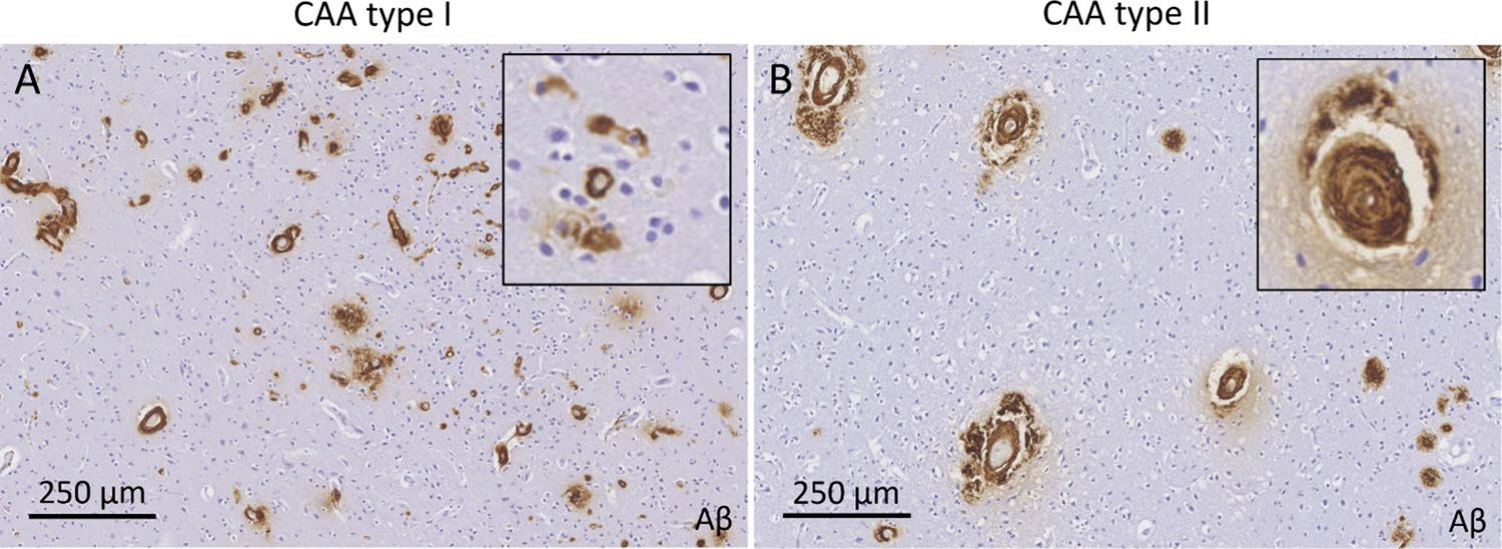
Examples from immunohistochemistry against Aβ in two autopsy cases with a clinical diagnosis of CAA, demonstrating extensive CAA with capillary involvement (**A**, CAA type I) and predominantly arteriolar involvement (**B**, CAA type II)

CAA is characterized neuropathologically by the presence of Aβ predominantly in the walls of cortical and leptomeningeal blood vessels, sparing the blood vessels in the white matter. Aβ first deposits in the media and adventitia, thereby gradually replacing the vascular smooth muscle cells [20]. Notably, endothelial cells appear to be relatively intact in CAA, suggesting that Aβ originates from the brain parenchyma rather than the systemic circulation, although a recent RNAseq study did find changes in human endothelial cell transcriptome in the presence of CAA [21]. Vascular Aβ deposition is typically first observed at the level of the leptomeningeal arteries, followed by the penetrating arterioles in the underlying cortex. Moreover, CAA progression tends to follow a posterior-to-anterior pattern whereby the occipital areas of the brain are affected earlier in the disease course. It has been suggested that the neuropathological patterns of Aβ deposition may reflect two distinct subtypes, where ‘CAA type I’ involves Aβ deposition in cortical capillaries, cortical arterioles, leptomeningeal arteries, and venules compared to ‘CAA type II’, where Aβ deposition is restricted to cortical arterioles and leptomeningeal arteries, sparing the cortical capillaries [22] (Fig. 1). In autopsy cases with capillary CAA (i.e. CAA type I), the frequency of the *APOE* ε4 allele is greater than in cases with predominantly leptomeningeal and arteriolar CAA (i.e. CAA type II) [22].

Advanced CAA is associated with the occurrence of numerous cortical microinfarcts [20, 23] and eventually may lead to vessel wall breakdown and hemorrhage [24]. Neuropathological studies found an association between arteriolar remodeling in the form of vessel wall splitting (Vonsattel grade 3) or fibrinoid necrotic changes (Vonsattel grade 4) and the number of hemorrhages in cases with severe CAA [24, 25]. Moreover, ex vivo MRI-guided targeted histopathological investigation of individual ruptured blood vessels suggested arteriolar remodeling as well as absence of Aβ at the site of rupture [26]. Accumulating evidence suggests that vascular remodeling may precede bleeding and that this process may in part be immune-mediated, which is accelerated in the context of CAA-related inflammation or anti-amyloid immunotherapy in the form of ARIA [4, 25, 27]. Whether perivascular inflammation and subsequent vessel wall breakdown may be triggered directly by vascular Aβ deposition or CAA-related subtle blood–brain barrier (BBB) leakage and plasma protein infiltration into the perivascular tissue remains to be determined. Furthermore, the mechanism underlying vessel wall rupture distant from sites of vascular Aβ accumulation remains unknown.

### Neuroimaging in patients with CAA

**Fig. 2:**
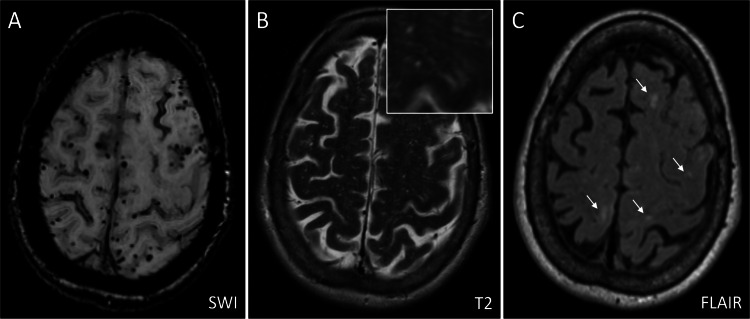
Traditional MRI markers of CAA include lobar cerebral microbleeds and cortical superficial siderosis (**A**). Recently, white matter features in the form of severe degree of MRI-visible perivascular spaces (**B**) and white matter hyperintensities in a subcortical multi-spot pattern (**C**, arrows) were added to the version 2.0 of the Boston criteria for the clinical diagnosis of CAA during life. SWI: susceptibility-weighted imaging, FLAIR: fluid-attenuated inversion recovery

Neuroimaging has a central role in the clinical diagnosis of CAA as provided by the Boston criteria, with the newest version published in 2022 [6]. A diagnosis of probable CAA can be made in individuals > 50 years of age who present with spontaneous ICH, transient focal neurological episodes, or cognitive impairment and have two or more strictly lobar hemorrhagic lesions (i.e. lobar cerebral microbleeds, ICH, or cortical superficial siderosis / subarachnoid hemorrhage) on blood-sensitive MRI scans. One of the up-dates made in the Boston criteria version 2.0 involves the addition of non-hemorrhagic MRI markers, in the form of a severe degree of MRI-visible perivascular spaces in the white matter centrum semiovale and white matter hyperintensities in a subcortical multi-spot pattern (Fig. 2). The presence of one of these non-hemorrhagic MRI markers in someone with one lobar hemorrhagic lesion increased sensitivity for the diagnosis of probable CAA, without compromising specificity [6]. Severe degree of MRI-visible perivascular spaces is defined as more than twenty visible perivascular spaces in the centrum semiovale of one hemisphere. The increased visibility of perivascular spaces on MRI has recently been described as potentially occurring in an earlier stage of CAA disease progression, prior to the observation of hemorrhagic brain lesions [5]. Of note, perivascular spaces are not specific for CAA as they can be found in many other settings as well, including cerebral small vessel disease involving arteriolosclerosis and AD [28].

Besides these findings on anatomical MRI scans, functional MRI scans have revealed more subtle disease processes that may occur in an earlier stage of CAA progression. By using functional MRI methods exploiting the blood-oxygen-level-dependent (BOLD) effect, the hemodynamic response can be measured as evoked by a visual stimulus. In sporadic CAA patients as well as in individuals with hereditary forms of CAA [29, 30], a smaller and delayed response has been observed by this approach as compared to age-matched control participants. Already in the pre-symptomatic phase of individuals with hereditary Dutch-type CAA, the BOLD response was found to be diminished. Moreover, after 5 years of follow-up, the BOLD response worsened in these participants, showing that stimulus-evoked BOLD functional MRI can, to some extent, be used to monitor disease progression [31].

### Pathological progression of CAA

Emerging insights from complementary studies in experimental mouse models, histopathological analyses of human brain tissue, and in vivo imaging studies in individuals with hereditary, sporadic, and iatrogenic forms of CAA have recently resulted in the presentation of a framework and timeline for the progression of CAA [5]. This observation-driven pathophysiological framework suggests that four key stages appear to evolve sequentially over the course of 2–3 decades. The first stage (stage 1) is represented by initial Aβ deposition in the walls of cortical arterioles as described above, followed by loss of physiological cerebrovascular responses (stage 2). Loss of vascular reactivity in response to visual stimulation has been observed in mouse models with CAA, where vascular Aβ depositions have replaced vascular smooth muscle cells [32], as well as in pre-symptomatic participants who carry the Dutch-type mutation for familial CAA [30]. Stage 3 is characterized by the occurrence of non-hemorrhagic brain injury, in the form of dilated perivascular spaces in the white matter centrum semiovale, white matter hyperintensities (predominantly in a subcortical multi-spot pattern), as well as cortical microinfarcts. In post-mortem human studies, these injuries have been associated with vessels in which Aβ has completely replaced the vascular smooth muscle cell layer, and the vessel lumen has narrowed (Vonsattel grade 2) [26, 33]. The final stage (stage 4) is when hemorrhagic brain lesions occur, including lobar cerebral microbleeds, cortical superficial siderosis, and ICH. Individual vessels associated with hemorrhagic brain lesions show signs of complete vascular remodeling in the form of Vonsattel grade 3–4 pathology [25, 26, 34]. From this framework it follows that patients who meet criteria for a diagnosis of probable CAA during life represent individuals with very advanced or end-stage CAA [6]. As such, initial vascular Aβ deposition which may be the result of impaired perivascular Aβ clearance seems to occur twenty to thirty years prior to when the disease can be recognized clinically. This has important implications for the development of novel intervention strategies, which will likely differ depending on which disease stage is targeted.

### Rodent models of CAA

Transgenic mice expressing neuronally-derived human *APP* harboring one or more familial AD (FAD) mutations have been the most commonly used experimental models to investigate CAA. For the most part, these models over-express human FAD mutant *APP* [35–38] and, in some cases, contain additional FAD mutations in presenilin proteins [39, 40]. Typically, these mouse models primarily develop parenchymal Aβ plaques followed by varying amounts of CAA, thus presenting with mixed cerebral amyloid pathologies. However, transgenic mouse models that express human APP containing one or more familial CAA mutations have been shown to specifically deposit vascular Aβ. For example, transgenic mice that express Dutch E22Q mutant Aβ in the brain develop robust CAA type II, although requiring aging to ≥ 24 months for the pathology to emerge [41]. On the other hand, the Tg-SwDI mouse line expresses chimeric Dutch E22Q/Iowa D23N CAA mutant Aβ in the brain and presents with striking capillary CAA (i.e. CAA type I) in a matter of several months [42]. These models provide paradigms to investigate the pathogenesis of CAA type I and CAA type II independently and in the absence of parenchymal Aβ plaques, although they have exhibited varying limitations in capturing the full human condition. More recently, a transgenic rat model for CAA type I, designated rTg-DI, produces chimeric Dutch E22Q/Iowa D23N CAA mutant Aβ in the brain like the Tg-SwDI mouse line [43]. The rTg-DI rats develop early-onset capillary Aβ deposition that captures many of the pathological features of human CAA type I including perivascular neuroinflammation, cerebral microbleeds, small vessel occlusions, white matter degeneration, and cognitive deficits [44–46]. Similarly, rTg-D rats were produced that express Dutch E22Q mutant Aβ in brain and capture many of the pathological features of human CAA type II with Aβ restricted to small arteries and arterioles and animals developing cerebral microbleeds, occluded small vessels, and cognitive decline [47]. This expanding collection of mouse and rat models of CAA offer opportunities for further mechanistic studies into the pathogenesis of this condition.

However, a significant shortcoming of all the aforementioned transgenic rodent lines is that the emergent CAA pathology develops from a ‘sole neuronal source’ of expression. Yet essentially all cells of the neurovascular unit express APP and can produce Aβ peptides. Thus, the complexity of the cellular origins of Aβ in CAA are not captured in ‘sole neuronal source’ transgenic models and likely do not reflect the true pathogenesis of this disorder. Recent advances in gene-editing in rodents offer the promise of providing more physiologically relevant platforms to model the true pathogenesis of CAA in humans. In this regard, new gene-edited rodent models are under development that express endogenous rodent APP, with specific amino acids substitutions in the Aβ domain, in a developmentally and spatially normal manner thus producing human CAA mutant Aβ at all physiologically relevant cellular sites. These novel gene-edited rodent models provide a unique opportunity to evaluate how CAA develops under more physiological settings that circumvent many of the artificial highly elevated expression and ‘sole neuronal sourced’ conditions of transgenics.

## Perivascular brain clearance

### Introduction and definitions

Perivascular spaces were historically known as Virchow-Robin spaces, after their anatomical description in the nineteenth century by Virchow and Robin [48, 49]. Insight into the functional relevance of the network of perivascular compartments, their adjoining vascular and parenchymal tissue components, is of much more recent debate. Thus, the contribution of perivascular spaces to brain clearance, and their connection to cervical lymphatic lymph nodes was suggested by the pioneering work of Cserr in the 1980s [50]. The significance of impaired drainage for Aβ deposition was noted by Weller and colleagues around 2 decades later [51]. In a landmark study by Iliff, Nedergaard and colleagues published in 2012 [52], the term ‘glymphatic system’ was introduced, describing a cerebral perivascular waste drainage pathway anatomically bounded by blood vessels and glial cells that supports the clearance of brain interstitial solutes to the CSF compartments. Functional coupling to the subsequently characterized meningeal lymphatic vasculature and cervical lymphatic drainage provides an integrated view of brain interstitial waste clearance [53, 54].

The initial glymphatic hypothesis included the centripetal influx of CSF along arteries, the advective movement of fluid and solutes through the brain interstitium that was dependent upon the perivascular astroglial water channel aquaporin 4, and the perivenous efflux of solutes into sinus-associated CSF compartments. However, a range of experimental observations centering on brain interstitial solute transport have resulted in active debate and ongoing revision of this concept. Carare, Weller, and coworkers introduced the ‘intramural peri-arterial drainage’ (IPAD) hypothesis that poses that while influx of CSF occurs along the peri-arterial channels, waste solutes do not exit via perivenous channels, but instead within the walls of the arteries retrograde to the direction of blood flow [55–58]. The other prevalent waste clearance concept, referred to as the ‘mixing model’, assumes that waste transport from the ISF is directed towards the peri-arterial channels, occurring along a concentration gradient (i.e. diffusion), and is facilitated by oscillatory ‘mixing’ from physiological motion (e.g. vasomotion and cardiac pulsatility [i.e. dispersion]) [59, 60]. The relative role and anatomical extent of bulk flow versus diffusive transport differs across these models, and likewise remains an important subject of current inquiry [61].

#### Box 1

Definition of the term ‘glymphatic system’ as used in this review = the network of perivascular channels and their adjoining vascular and parenchymal tissue components that function to facilitate the exchange and clearance of the brain’s waste solutes carried in the interstitial fluid. Thus, it is a working model that encompasses not only the traditional glymphatic hypothesis, but also the IPAD and mixing hypotheses.

### Current understanding and unknowns

There is a lack of consensus on the anatomy and directionality of periarteriolar transport pathways; meaning the precise location of solute transport within or next to the arteriolar wall, and whether this occurs antegrade or retrograde with respect to blood flow. At the level of larger vessels, fluid and solute transport may occur along basement membranes of smooth muscle cells in the arteriolar wall versus perivascular transport adjacent to the wall (i.e. between the outer layer of smooth muscle cells/adventitia and the glia limitans). One possibility is that both pathways exist as outflow and inflow routes respectively (as suggested by the IPAD model [62]). Alternatively, there is only one pathway, but methodological differences have led to different interpretations and thus controversies [63]. Important methodological considerations here are the location and intensity of tracer injection (into CSF versus intra-parenchymal), molecular weight and composition of the tracer, timing, and in vivo versus post-mortem analysis. Careful experimental approaches are needed here to provide a definitive answer to this question. A systematic analysis of tracer transport, comparing different sizes of molecules, inert versus biologically active tracers, time points, and one-to-one comparison of in vivo to post-mortem data could resolve some of these uncertainties.

Notwithstanding the precise anatomical route of drainage along arteries, few data on perivenous spaces and flow have been reported, and even less on pericapillary spaces. In humans, most MRI-visible perivascular spaces were found to be around arteries [33, 64, 65]. Assuming that perivascular spaces are visible on MRI when they are pathologically dilated only, this could mean that perivascular spaces around veins are less prone to dilation. The reasons for this are open to speculation. Perhaps the venous side of the circulation is less affected by vascular pathology, including CAA and BBB dysfunction, which could play a causal role in dilation of the perivascular space. In addition, the biophysical environment may differ between periarterial and perivenous spaces, which includes the local pressure gradients and flow profile. Further studies are needed to provide data on these issues. A second question about perivenous spaces concerns their role in drainage of fluid and solutes. Studies using multiphoton imaging of mouse brains have shown pulsatile perivascular flow along pial arteries [66, 67], but there is a paucity of such data regarding perivenous flow. There are several reasons as to why it has been much harder to delineate the interstitial efflux routes, including drainage along perivenous spaces. First, upon infusion of tracers directly into the interstitium, physiological routes are easily overwhelmed by even small injection volumes. Second, when injected into the CSF, tracers are diluted upon entry of the large volume of the brain interstitium after their entry along periarteriolar spaces. The timing of tracer efflux is another possible explanation. According to the first studies on the glymphatic system [52], perivenous outflow of tracers from the interstitium may become apparent a few hours after influx, which may be beyond the time frame of most studies. Notwithstanding these technical difficulties, tracers injected into the interstitium leave the brain faster towards the CSF and lymphatic system than to be expected from diffusion alone. This occurs along preferential pathways, including perivascular spaces as well as white matter tracts. The contribution of periarteriolar versus perivenous spaces in soluble waste drainage, and how these differ upon anatomical location, physiological, or pathological condition are part of an area of active research.

The connection between perivascular spaces at the transition between the leptomeningeal and cortical segments of the vessels deserves specific attention. Studies have shown that small and larger molecules, but not microspheres (e.g. fluorescent polystyrene particles of ~ 1 μm in size), enter the perivascular space from the pial surface further along penetrating vessels [66, 67]. In parallel, post-mortem high resolution anatomical data in the human brain show pial sheets at this location [68]. The functional relevance of these pial sheets is currently unknown, but they may prohibit the entry of larger particles, cells, and infused microspheres, possibly acting as a kind of sieve, depending on the anatomical location. The lack of microsphere penetration along the perivascular spaces of penetrating vessels limits the possibility for direct observational studies using this approach. Alternative methods are therefore needed to determine the flow patterns along penetrating vessels.

Besides the anatomical uncertainties, other fundamental unknowns include the driving forces of the glymphatic system. Vascular pulsatility, originating from the cardiac cycle, was one of the first proposed driving forces [1]. Indeed, microspheres were observed to move in a pulsatile manner at the brain surface at the frequency of the heartbeat [66, 67]. In line with this hypothesis, conditions associated with impaired vascular pulsatility, such as aging [69] and acute hypertension [66], showed impaired glymphatic function. Yet, while there is little discussion on whether vessel oscillations promote glymphatic function, there is ongoing debate on the question of whether they induce a net perivascular flow, rather than a to-and-fro movement. Modeling studies have not reached consensus on this point [60, 70–73].

In addition to cardiac pulsatility generated by the heartbeat, other sources of vessel oscillations may promote glymphatic function. Respiration affects CSF motion in the human brain [74], but the impact on glymphatic function is not well studied. However, a study in spontaneously breathing rats showed that augmentation of respiratory function by continuous positive airway pressure promoted CSF as well as glymphatic transport [75]. As respiratory oscillations mostly affect the venous side of the circulation, this is an interesting subject for further study [74]. More recently, vasomotion emerged as an important driver both in the traditional glymphatic and in the IPAD concepts. Vasomotion refers to large arteriolar dilations that are generated by smooth muscle cell contractions, which can occur spontaneously in isolated perfused arteries and is believed to be entrained by neuronal activity in the living mouse brain [76]. Driving vascular reactivity at the intrinsic low vasomotion frequency using non-invasive sensory stimulation was found to increase clearance of local dye extravasation in mice [32, 77]. As the changes in diameter during vasomotion are relatively large (in comparison to pulsatile oscillations induced by the heartbeat), the volume of perivascular fluid that oscillates is also relatively large. Interestingly, vasomotion is synchronized during certain stages of sleep, predominantly during non-rapid eye movement sleep. This is associated with brain wide oscillations in CSF motion that are anti-correlated with blood flow oscillations, implying that when cerebral blood volume increases, CSF is pushed out of the cranium [78]. Thus, it is very attractive to hypothesize that synchronized neuronal activity, blood flow, and CSF motion contribute to brain clearance during sleep [79].

#### Box 2

Advection is the overall motion of a fluid, characterized by the average (barycentric) velocity of its components, often referred to as the ‘bulk flow’. Advection also denotes the transport of any quantity in this fluid (e.g. waste concentration, heat) by this overall motion.

Molecular diffusion is the macroscopic manifestation of Brownian motion, by which concentration gradients tend to homogenize the composition of the medium according to Fick’s law. Besides free diffusion in CSF, diffusive processes also include facilitated diffusion across cell membranes, e.g. by channels or carriers [80] and effective diffusion, resulting from the interplay between molecular diffusion in the different components of the parenchyma (ISF, cytosols, cell membranes) and its microarchitecture.

Diffusive processes superimpose on advective motion, and their combination can lead to dispersion when heterogeneous velocity fields bring into proximity fluid with different concentrations, resulting in faster homogenization than by diffusion alone due to increased concentration gradients.

## Evidence for CAA as a glymphatic system disorder

### Human neuropathology studies

**Fig. 3:**
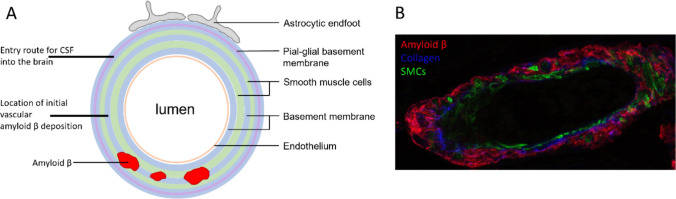
Schematic of a human brain arteriole with the proposed entry route for CSF into the brain as well as the location of initial vascular Aβ depositions indicated (**A**). An example of an (X40 SP8) confocal microscopy image of a cross-section of a human brain arteriole with CAA, stained for Aβ (red), collagen IV (blue), and laminin (green) (**B**). Note that the Aβ is surrounding the smooth muscle cells in tunica media. SMCs: smooth muscle cells

Human post-mortem studies show that the distribution of Aβ in the walls of capillaries and arteries is along the basement membranes surrounding intramural cells [56]. There are distinct patterns suggesting progression from Aβ deposition in the central part of basement membranes, to complete co-localization of Aβ with basement membranes in tunica media, leaving the endothelial and glia limitans basement membranes free in the initial stages of disease [81] (Fig. 3). This suggests that the direction of drainage of Aβ from the parenchyma is from the capillary basement membranes into the basement membranes of tunica media rather than influx from the CSF, as that occurs along the glial-pial compartment [57].

In brain tissue of patients with hereditary transthyretin amyloidosis and central nervous system involvement, misfolded transthyretin (TTR) was found to accumulate in the leptomeninges [82]. In these patients, deposition of TTR, which is primarily produced peripherally and by the choroid plexus, follows a pattern whereby leptomeningeal vessels are affected earlier, then followed by deposition in the walls of penetrating cortical arterioles, and subpial deposition. As amyloids are first introduced in the CSF compartments in this disease, rather than the parenchyma, it may provide an interesting ‘model’ to study amyloid deposition and distribution patterns that follows traditional CSF glymphatic influx pathways.

Another source of human neuropathological data that has been informative for understanding glymphatic system dynamics has come from brain tissue of AD cases that were part of the first Aβ immunotherapy trials [83]. Active immunization against Aβ led to the reduction in the number and surface area of Aβ plaques, but a worsening of CAA, suggesting that Aβ is entrapped in its perivascular drainage pathways after solubilization from plaques [84]. Furthermore, Aβ immunotherapy was associated with redistribution of ApoE from cortical plaques to cerebral vessel walls, mirroring thus the pattern of distribution of Aβ_42_ from plaques to the vessel walls [85]. As such, the human neuropathologic distribution of vascular Aβ suggests that it is deposited in the pathways suggested by the IPAD model present in the tunica media of cerebral arteries.

### Rodent studies

There is a growing body of literature derived from rodent imaging studies suggesting that the glymphatic system facilitates perivascular Aβ transport and that the accumulation of vascular Aβ in the form of CAA interferes with this system. Rodent studies investigating perivascular soluble Aβ movement rely on the injection of tracers into the CSF- or ISF-containing spaces of the brain, followed by real-time in vivo or single time point ex vivo imaging of tracer influx or efflux. Studies in wild-type mice that have relied on injection of tracers into the cisterna magna, have consistently found tracers to enter the brain primarily via periarteriolar rather than perivenous spaces [52, 86]. Similarly, fluorescently tagged Aβ_40_ that was injected into the mouse cisterna magna entered the brain via periarteriolar spaces [52, 86]. Real-time in vivo multiphoton microscopy imaging has revealed that small-molecular weight tracers readily move into the interstitium, whereas larger (~ 2kD) tracers remain confined to the periarteriolar space. Likewise, Aβ_40_ (which is the predominant species depositing in CAA) influx from CSF along periarteriolar spaces appears to be more evident compared to Aβ_42_ (which is primarily found in parenchymal plaques) [86]. As also mentioned above, although it has been suggested that intra-cisternally injected tracers that enter the brain along arteries are cleared along veins, there is paucity of real-time in vivo imaging data supporting this claim.

To study clearance pathways of Aβ directly from the interstitium, which is the site of endogenously produced Aβ, studies have adopted intra-parenchymal (e.g. in cortex, striatum, or hippocampus) injection strategies, followed by ex vivo imaging of tagged Aβ patterns [52, 57, 87, 88]. Radiolabeled ^125^I-Aβ_40_ was found to be cleared from the interstitium rapidly, with more than half of the injected dose being eliminated within 30–60 min [69, 87]. Clearance rates appear to be reduced in old mice [69], whereas sleep, ketamine/xylazine anesthesia or balanced anesthetics with dexmedetomidine may improve clearance [87, 89]. Studies that directly examined the anatomical pathways of Aβ efflux found that tracer injected into the parenchyma primarily ended up in the walls of capillaries and arterioles, specifically in the basement membranes surrounding vascular smooth muscle cells [57, 88]. Interestingly, one study found a shift from tracer localization from the capillaries and arterioles to the venules in aged mice with CAA. Dextran labeling of venules was found to be increased proportional to CAA severity in very old Tg2576 mice, such that 22-month-old mice demonstrated significantly more labeling of venules compared with young or adult mice [88].

Both tracer influx [86, 90] and efflux have been observed to be impaired in transgenic mice that produce mutant human Aβ [32, 86, 88, 91]. Moreover, genetic deletion of aquaporin 4 resulted in a significant reduction of Aβ_40_ influx or clearance [52], suggesting that these water channels on astrocytic endfeet facilitate Aβ transport in the brain [52, 69, 92].

Real-time multiphoton microscopy studies have helped elucidate the driving forces of perivascular brain clearance in vivo. As noted above, vascular pulsations have been implicated early on in facilitating tracer movement along perivascular spaces [52, 69, 91]. Real-time tracking of fluorescent particles that were injected into the cisterna magna of anesthetized mice demonstrated that CSF flow along pial arteries is pulsatile and driven by beat-to-beat arteriolar pulsations generated by the cardiac cycle [66]. More recently, studies in unanesthetized mice pointed at vasomotion as another likely candidate to facilitate tracer movement along periarteriolar spaces [32, 93]. Relying on the principle of neurovascular coupling, visual stimulation at the innate vasomotion frequency centered around 0.05 Hz in mice, was found to significantly increase vasodilation as well as tracer clearance from the perivascular space at the pial surface in wild-type mice [32]. Of note, visually evoked vascular reactivity was found to be impaired in transgenic mice with CAA, which corresponded to impaired tracer clearance along CAA-positive vessels in these animals [32]. In line with these findings, a recent study found that tracer influx was significantly increased during whisker stimulation-induced functional hyperemia [93]. Moreover, when directly stimulating the vascular smooth muscle cells utilizing optogenetics, thereby circumventing neurovascular coupling, tracer influx was similarly found to be enhanced compared to no stimulation [93]. These findings suggest that smooth muscle cell-mediated impedance pumping may be a key driver for facilitating solute movement including Aβ in the brain.

Glymphatic solute transport and clearance was recently investigated in a transgenic rat model of CAA type I using dynamic contrast enhanced MRI (DCE-MRI) combined with intracisternal administration of a Gd-tagged tracer [94]. The study was carried out in a CAA type I transgenic rat (rTg-DI) line. Re-routing of high-speed subarachnoid CSF flow currents away from the brain was observed, resulting in a decline in glymphatic transport as well as aberrant solute clearance to extracranial lymph nodes. Thus, in the rTg-DI rats, solute drainage to the deep cervical lymph nodes was time-delayed with ‘spill-over’ outflow to the accessory lymph nodes [94]. These results in CAA rats are in stark contrast with results obtained in DCE-MRI studies in rat models of cerebral small vessel disease with chronic hypertension (of note, the rTg-DI CAA rat model is not hypertensive). For example, in spontaneously hypertensive stroke prone rats (SHRSP) with chronic hypertension, CSF flow is ‘sluggish’ and associated with decreased glymphatic transport implying that vascular pulsatility, and tracking of CSF flow are important metrics to capture for interpreting the pathophysiology of glymphatic-lymphatic system transport in neurodegenerative disease states.

### In vivo* human studies*

Apart from the neuropathological findings, supporting evidence that CAA pathophysiology is related to changes in brain clearance stems from measurements in CSF samples. Similar as what has been observed in patients with AD, in the CSF of CAA patients Aβ_40_ and Aβ_42_ are both reduced, corresponding to an increased build-up of these peptides in the brain itself [95]. In individuals with Dutch-type CAA, Aβ concentrations in CSF were found to be decreased in a group of five pre-symptomatic mutation carriers compared to age-matched controls and this concentration decrease was more severe than in sporadic CAA [96]. Moreover, it was noted that the decrease in Aβ_42_ was larger than Aβ_40_, which might be indicative that accumulation of Aβ_42_ precedes that of Aβ_40_ [97], although these findings need to be replicated in larger samples Similar findings have been obtained in blood plasma [98].

From a neuroimaging standpoint, the occurrence of MRI-visible perivascular spaces in the brains of CAA patients stands out [5]. MRI-visible perivascular spaces in the centrum semiovale were associated with locally increased CAA severity in the cortex measured post-mortem [33] and with increased Aβ Positron Emission Tomography (PET) positivity measured in vivo [99], suggesting a direct link between impaired Aβ clearance and enlarged perivascular spaces. Dilation of perivascular spaces may be a secondary effect of Aβ deposition in cortical arterioles, resulting in impaired perivascular fluid flow in downstream connected compartments. Other potential mechanisms that required further research include direct effects of white matter tissue injury [33] or the presence of soluble Aβ within the white matter of the brain [100].

With respect to the driving forces that might influence the efficiency of glymphatic function, the most studied process in the human brain is vasoreactivity. Impaired vasoreactivity has been hypothesized to decrease glymphatic function due to stiffening and decreased mobility of the vessel wall. Both in sporadic CAA [29] and in Dutch-type CAA [30], the response to a visual stimulus was found to be delayed and decreased. Moreover, these signs of dysfunction of the cerebral vasculature worsened during a 4-year follow-up in Dutch-type CAA [31]. This might induce a self-amplificatory cycle: due to impaired clearance Aβ starts to accumulate in the vessel wall, the wall becomes stiffer and less vasomotion will occur, which impairs clearance even further, leading to even more Aβ accumulation. As a true cycle it is of course unknown where this process starts, and this should be an important future research point to elucidate.

## Perspectives for future research / knowledge gaps

### Anatomy

**Fig. 4:**
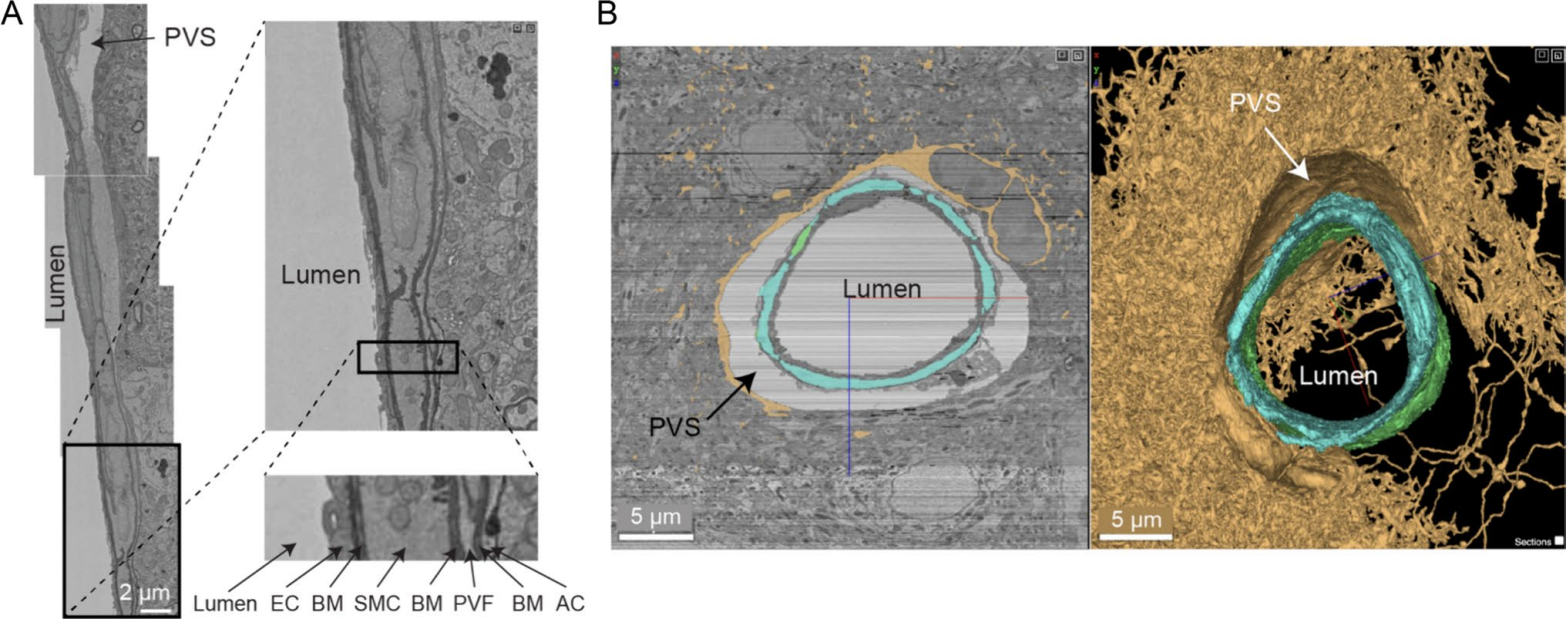
The wall and perivascular space of a penetrating arteriole from the MICrONS Cortical MM^3 volume EM dataset (**A**). Magnified views of the arteriole wall showing the perivascular space diminishing in size and becoming compact layers of different vascular cell types with greater cortical depth (pial surface is toward the top of the image). Specifically, the perivascular space appears to become continuous with the perivascular fibroblast layer and the basement membrane that flanks these cells. EC: endothelial cell, BM: basement membrane, SMC: smooth muscle cell, PVF: perivascular fibroblast, AC: astrocyte. Adapted from [101]. 2D cross-section and volume rendering of penetrating arteriole from mouse cortex showing ultrastructure of perivascular compartment (**B**). Yellow = one astrocyte. Blue/green = two individual smooth muscle cells

Understanding the anatomy of the perivascular pathways for glymphatic clearance poses unique challenges. It requires imaging of the vasculature at nanometer resolution to visualize vascular wall structure, but over hundreds of micrometers to understand differences in vascular structure along the arteriole-capillary-venous axis. Recent efforts to map neural circuitry on a large scale using serial block face electron microscopy (EM) have demonstrated the feasibility of creating such datasets, but the complexity in creating and analyzing these data is substantial [101]. In particular, the Cortical MM^3 dataset, created by the MICrONs consortium, contains a cubic millimeter of adult mouse visual cortex imaged at ultrastructural resolution [102] (Fig. 4). The data contain extensive information on microvascular structure, including all neurovascular cell types, perivascular spaces, and the layers of basement membrane that envelop the vascular cells. It provides a glimpse into what can be achieved with future efforts to map the meningeal and perivascular routes in 3D.

Future work could attain quantifiable 3D morphometrics of neurovascular cells, spaces, and non-cellular components from all zones of the microvascular network and develop segmentation approaches for automated detection of these structures. This information is needed to understand the flow routes and barriers or resistance points of CSF flow, which may not be identifiable from localized 2D imaging. For example, there remains heavy debate on the anatomy of the meningeal layers [103–105], which contains perivascular routes for CSF perfusion within the subarachnoid space. Yet, there is limited knowledge on how these meningeal perivascular networks are organized, and how they connect with perivascular spaces of the penetrating arterioles. As discussed above, some observations suggest the existence of pial sheets that could restrict CSF flow between the subarachnoid and perivascular spaces. Do these sheets create CSF flow resistance, and are they found on all penetrating arterioles? Further, the size and location of fibroblasts and perivascular macrophages within the perivascular spaces would also contribute to CSF flow resistance and should be understood more deeply for accurate modeling of CSF transport. The dimensions and continuity of the basement membrane ensheathing the vessel wall is also necessary to understand in 3D, as presumably this creates the route for CSF flow as perivascular spaces diminish along smaller brain arterioles. The coverage of astrocytic endfeet also remains poorly characterized yet vital to understanding whether fluid exchange with the CSF-ISF can occur through spaces between tiled astrocytic endfeet, in addition to aquaporin 4-mediated fluid exchange at this level. Critically, as discussed below, these anatomical data should be characterized in normal and CAA tissues and linked with physiological measurements of CSF flow along specific perivascular spaces using new in vivo imaging approaches.

Many initial insights were made from qualitative surveys of MICrONS Cortical MM^3, which was obtained from a normal adult mouse brain. For example, perivascular spaces are discernable for some but not all cortical penetrating arterioles, and only in the upper cortical layers. The perivascular space is bounded by pial and endothelial basement membranes and contains perivascular fibroblasts and macrophages [106]. The space gradually disappears and becomes a densely packed wall with endothelial, mural, fibroblast/macrophage, and astrocytic layers embedded within the basement membrane [101]. As smaller microvessels branch from penetrating arterioles, there is no discernable perivascular space around arteriole-capillary transitional zones or capillaries, but the basement membrane itself may serve as a porous medium for transit of CSF [107]. Perivascular spaces are also not seen along cortical venules either, though there is a layer of basement membrane. The quantification of key anatomical features, such as the dimensions of the perivascular spaces and the thickness and continuity of the basement membrane, will be important for efforts to model CSF flow.

The application of volume EM techniques in rodent models of CAA will enable a deeper understanding of how CAA alters the neurovascular unit and perivascular space. Blood vessels occupy a small proportion of the total brain tissue volume, and imaging strategies to broadly scan and then collect volume EM from specific vascular regions of interest will be needed to capture heterogeneous CAA pathology across vessels [108]. Electron-dense tracers that can be detected by EM can be injected into the cisterna magna to visualize where CSF is able to carry solutes in the perivascular compartments. These efforts will provide insight on how CAA-induced arteriolar tortuosity and widening of perivascular spaces are related to cellular/subcellular level changes in neurovascular cell types and basement membrane integrity. In humans, enlarged perivascular spaces are more prevalent in the cerebral white matter. Specific comparison between gray and white matter should be made to understand why some tissues are more strongly affected. Decisions will need to be made on which models would best align with clinical data. Several rat and murine models express human APP with different mutations that result in more Aβ deposition in the cerebral arterioles to capillaries, which may help distinguish features of CAA type I versus CAA type II observed in humans.

The caveats associated with tissue fixation and preservation of extracellular spaces considered in mapping of neural circuitry would also apply to vascular/perivascular compartments. It will be important to determine if typical fixation and processing for volume EM affects perivascular space dimensions, due to tissue shrinkage or distortion. This may require in vivo multiphoton imaging in the same samples/regions used for volume EM so direct comparison can be made at the level of individual vessels. Further, studies will be needed to determine whether fixation procedures affect the distribution of tracers introduced into the CSF.

### In vivo* mouse imaging studies*

In vivo multiphoton microscopy and MRI have been the main approaches used to study the dynamics of brain clearance in mice. As discussed above, typical studies involve measuring the clearance of exogenous tracers injected into the brain tissue or following intracisternal injection to label the CSF. Fluorescent dyes are also injected into the cisterna magna to fill the perivascular space for structural imaging or to track the movement of small fluorescent beads in perivascular regions on the pial surface. Multiphoton studies involve the generation of cranial windows for high-resolution, local visualization of dye in mouse cortex, whereas MRI studies have typically used rats for their larger brain size. Disparities between mouse and rat model systems, let alone rodent versus human systems, are not well understood making it potentially challenging to relate multiphoton and MRI data in the future, in addition to the distinct spatial scales being examined.

Best practices for studying glymphatic clearance using in vivo imaging are still being honed, and the key concerns are how anesthetics, surgical procedures, and dye injection procedures affect CSF flow and solute transport. Early studies were performed under anesthesia and showed that ketamine/xylazine or isoflurane/dexmedetomidine combinations were best for accessing the perivascular spaces following intracisternal injection [109]. There is a shift toward imaging unanesthetized, head fixed mice, as this would best capture native CSF flow dynamics and allow imaging during sleep states, which can be tracked by monitoring of brain activity or pupil diameter [110]. However, imaging unanesthetized rodents remains challenging for MRI studies, and some sedatives may serve as alternatives [111]. Multiphoton imaging studies require creation of a cranial window for optical access. However, little is known about how changes in intracranial pressure, tissue compression and inflammatory reactions induced by surgery affect CSF flow. Less invasive, thinned skull cranial windows could be a complementary approach in mice to verify key findings from studies that use conventional skull-removed windows.

**Fig. 5:**
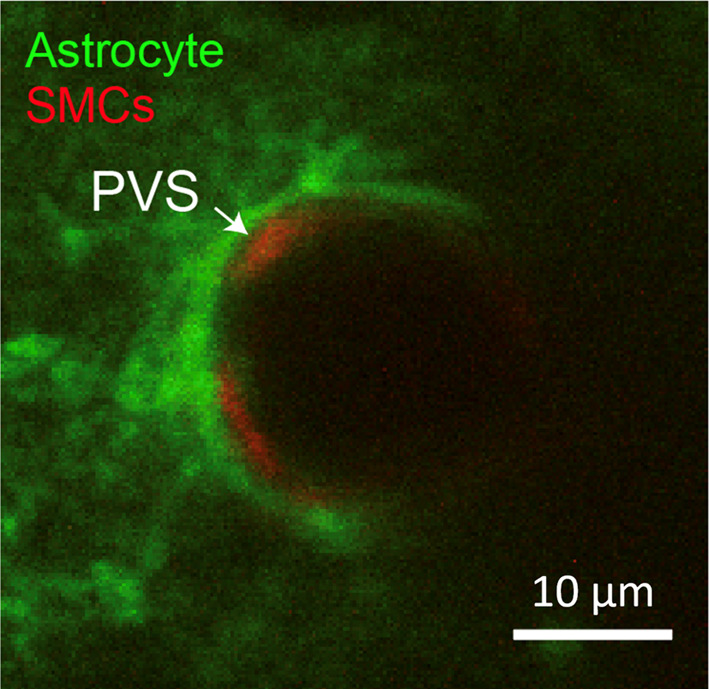
Visualization of penetrating arteriolar perivascular space bounded by astroglial endfeet and vascular smooth muscle cells in vivo using multiphoton microscopy. Images were taken in an isoflurane anesthetized mouse. Astrocytes (green) are labeled by AAV-GFAP-Lck-GFP and smooth muscle cells (SMCs; red) are endogenous to PDGFRβ-tdTomato mice

Future studies should also try to overcome the limitations of acute tracer injection for glymphatic clearance studies. For example, studies have shown that acute implantation of a small cannula or needle can suppress CSF flow dynamics and glymphatic transport [112]. More recent ex vivo studies have suggested that the acute tissue trauma from a needle stab or implantation to inject tracer may be ameliorated by increasing the time from cannula implantation to testing of glymphatic transport [113]. For intra-parenchymal and intra-cisternal injections, volumes should be kept as small as possible to reduce increases in intracranial pressure and tissue injury. Another fundamental limitation is that focal injections of fluorescent tracers create an artificial diffusion gradient from the point of injection. To differentiate between diffusive and advective tracer movement, and to minimize acute responses to tissue injury, it may be possible to chronically deliver photo-activatable tracer molecules into the CSF and ISF through osmotic pumps. This would allow a steady state level of tracer to be first established throughout the brain, and the clearance/flow direction of photo-activated dye could potentially be tracked in the tissue parenchyma or perivascular space. Further, it will be necessary to understand how the volume of perivascular spaces change with physiological rhythms in awake rodents (cardiac pulsatility, respiration, vasomotion). However, this would ideally be studied without exogenous tracer injections that could change the volume or the viscosity of the CSF. Transgenic or viral approaches to fluorescently label cells that flank the perivascular space (astrocytes, smooth muscle cells) may be a way to study dimensions of the pericyte space noninvasively (Fig. 5).

### Human imaging of glymphatic clearance

With impaired glymphatic clearance as a relatively recently proposed pathophysiological mechanism implied in CAA and other neurodegenerative diseases, the ability to measure perivascular clearance efficiency in the human brain is becoming increasingly important. Whereas most of our knowledge on glymphatic clearance is derived from rodent models in which invasive techniques such as multiphoton microscopy through cranial windows can be applied, our ability to measure, let alone quantify, brain clearance in living human beings is limited. The current gold standard for human brain clearance imaging is based on intrathecal injections of MRI contrast agent and subsequent dynamic T1-weighted MRI at intervals of 3–6 h over a period of 2 days [114–116]. These experiments have resulted in a significant amount of information on the glymphatic system and highlighted important differences between humans and rodents. However, two important drawbacks will impede widespread use of this approach. First, it relies on intrathecal injections, and although the number of side-effects as well as retention of contrast agent in brain tissue, have been very mild, it is still considered a highly invasive procedure [117, 118]. Moreover, it relies on off-label use of the contrast agent. Secondly, these measurements have a very long temporal footprint, as the contrast agent first needs to flow into the brain, enter the brain tissue, and subsequently be cleared. This implies that measurements are done over 24–48 h, which present another hurdle for wide-spread use. Although intravenous injections have been proposed as a more patient-friendly alternative, these experiments also rely on a long temporal footprint and interpretation of the data is complicated, because delivery into the tissue will be dependent on the integrity of the BBB, or entrance of contrast agent via other routes (such as into the subarachnoid space).

Therefore, there is a need for non-invasive techniques to measure brain clearance. Several approaches are being developed with most of them relying on measuring CSF mobility at different levels of the glymphatic system. The general argument is that many waste products are soluble in water and that CSF is the conduit that will transport these out of the brain. Fultz and Lewis studied CSF mobility and the relationship with vasomotion, as measured by the coupling of low-frequency BOLD fluctuations in the cortical gray matter with CSF inflow in the fourth ventricle [78]. Importantly, they also studied these phenomena during sleep with MRI-compatible EEG to monitor the sleep stage. They show that CSF inflow in the fourth ventricle is linked to low-frequency oscillations in brain activity and that these physiological processes are enhanced during sleep. The same approach also showed that this coupling is disturbed in AD [119]. An important difference with the contrast agent approaches is that these measurements look at interactions between vasomotion activity and CSF-mobility with a total scan duration of 5–10 min. Such a scan (which is similar to a standard resting state functional MRI scan that is planned with the lowest slice through the fourth ventricle) can be easily inserted in clinical studies or be used to monitor changes across time. However, the fourth ventricle is quite far from the location where Aβ is produced or where it accumulates in CAA. Therefore, other methods are being developed that can measure CSF mobility closer to the site of endogenous Aβ production. For example, by exploiting the longer T2 of CSF, the CSF signal can be isolated by using a long echo time. This approach has both been exploited in rodent models as well as human studies [120, 121]. Finally, there are recent studies measuring brain tissue deformation during the cardiac and respiratory cycle, with the assumption that these deformations may contribute to driving the clearance systems of the brain [122, 123].

Of note, arguably the most applied technique at the moment is the diffusion tensor imaging along the perivascular space (DTI-ALPS) analysis that can be performed on rather standard DTI-scans [124]. The concept is based on perpendicular orientation of perivenous spaces to major white matter tracts in a region next to the ventricles. One study has looked at the application of DTI-ALPS in CAA and found a decreased DTI-ALPS index, and correlations of this decrease with enlargement of perivascular spaces in the basal ganglia, the severity of white matter hyperintensities, and cognitive impairment [125]. It should be noted that there is an ongoing discussion on whether DTI-ALPS is an accurate measure of glymphatic activity [126]. This analysis has been criticized for only focusing on a small region in the brain, for not isolating the CSF signal, and the fact that the orientation of the perivenous spaces is not checked in many studies. Results should therefore be interpreted with caution.

### Rodent MRI as a bridge to human imaging

**Fig. 6:**
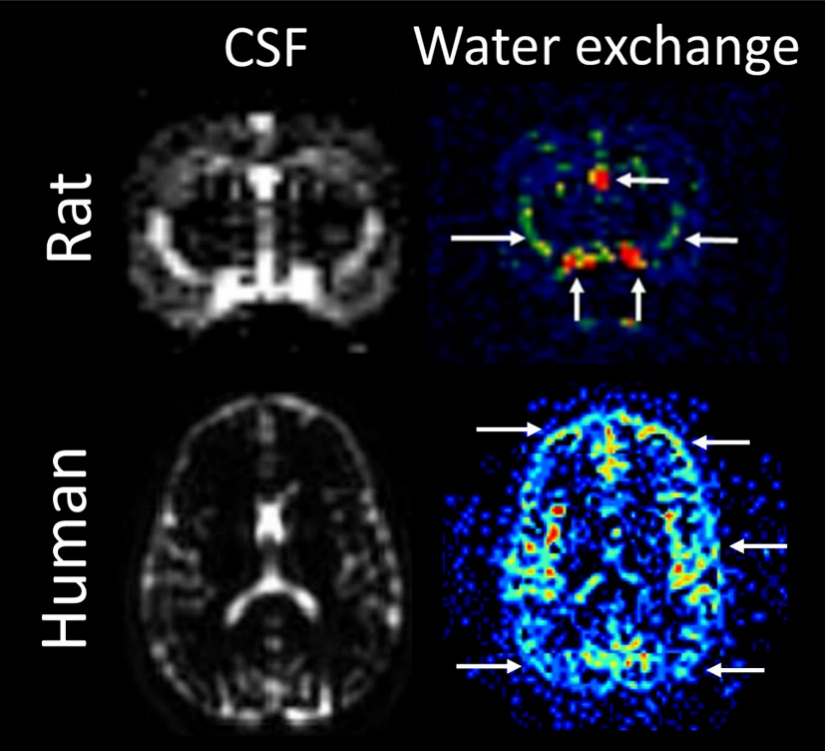
Arterial Spin Labeling (ASL) is a suitable translational MRI technique that non-invasively captures blood-to-CSF water exchange in the rat and human brain. By employing long labeling durations, long post-labeling delay times and a long echo time, the arrival of labeled spins in the CSF can be captured. The long echo time ensures that signal from brain tissue is already nulled, leaving only signal in water-like regions, like ventricular and cortical CSF. Note that exchange is not limited to the ventricles but is also found around the cortex

Dynamic contrast enhanced MRI (DCE-MRI) combined with CSF administration of a Gd-tagged tracer was introduced as a preclinical imaging method to investigate glymphatic transport in the rodent brain [127, 128]. This approach captures glymphatic transport when the CSF tagged with Gd-tracer enters the brain parenchyma over 2–3 h in a distinct spatial distribution pattern. Specifically, rodent DCE-MRI studies visualizes two major ‘waves’ of Gd-contrast entering into the brain: a ‘ventral’ wave moving towards the olfactory bulb and a ‘dorsal’ wave moving towards the pineal recess [127]. Regionally the ventral wave includes the brain stem, hypothalamus, frontal and olfactory cortex, while the dorsal wave incorporates the cerebellum, occipital and retrosplenial cortex, hippocampus, and midbrain [129]. This particular pattern of Gd-tracer uptake in the rodent brain has been reproduced in several other animal species including non-human primates [130], canines [131] and pigs [132] as well as in the human brain [133]. So far, the DCE-MRI based studies of glymphatic transport in rodent and human brain have yielded largely similar results: (1) the brain-wide distribution pattern of Gd-tracer influx into brain parenchyma is similar albeit with different time-scales [128, 134], (2) Gd-tracer influx from subarachnoid CSF occurs along the penetrating arteries, (3) Gd-tracer transport in the brain parenchyma is regionally heterogeneous, (4) clearance of the Gd-tracer from brain parenchyma requires several hours [134], and (5) the Gd-tracer drains to the cervical lymph nodes [94, 135]. Although DCE-MRI has become a ‘gold standard’ imaging technique for measuring brain-wide glymphatic transport it is invasive and not practical for routine use in humans. As mentioned above, less invasive MRI techniques continue to be introduced to further explore CSF flow dynamics in humans, some of which originate from rodent MRI. For example, Harrison and colleagues measured CSF mobility around the middle cerebral artery with high spatial resolution diffusion-weighted MRI which was subsequently also proposed in humans [120]. However, given that there is no definite measure of ‘solute’ transport with any of these techniques besides water there is still an urgent need to cross-validate the surrogate MRI metrics now referred to in the literature as ‘glymphatic transport’ in the human brain with preclinical studies (Fig. 6).

According to the glymphatic system model, the bulk-flow driven peri-arterial CSF transport facilitates CSF-ISF mixing and waste removal via perivenous channels. Other studies have further demonstrated that the glymphatic system is coupled to the meningeal lymphatics (mLV) and that the perivenous outflow of ‘dirty’ brain fluids collects into the mLV that drain primarily to the deep cervical lymph nodes (dcLN) [53, 54]. Surgical approaches (e.g. ligation of the afferents to the dcLN or excision of dcLN) to uncouple the glymphatic system from the lymphatic drainage pathways have revealed acceleration of neurodegenerative pathology in certain rodent models [136]. However, the mechanisms underlying the negative impact of glymphatic-lymphatic system uncoupling on brain health remains incompletely understood and controversial [137].

### Develop novel integrated computational models

As studies have sought to define perivascular transport and glymphatic function, computational modeling has emerged as a key tool in defining this biology and beginning to understand the ways in which it is regulated under physiological conditions and becomes mis-regulated in pathological states. For a more complete discussion of the application of computational modeling to the study of glymphatic function, the reader is directed to a recent review by Bohr and colleagues [138]. In brief, approaches to computational modeling can serve several useful ends as we try to understand the processes governing brain waste clearance.

First, models can serve as a generalized framework where data of different types and from distinct sources can be integrated to inform biological processes. For example, three different recent imaging and histological studies have reported direct measurements of the shapes and sizes of perivascular spaces at the pial surface [139], flow velocities within these perivascular spaces [66] and perivascular astroglial endfoot dimensions within the cortex [140], aspects that are anticipated to influence perivascular fluid and solute transport. While each study was conducted in isolation, and their results are not directly inter-relatable, computational models provide a medium for integrating these observations and understanding their respective and combined influences on fluid and solute transport along perivascular compartments. For example, a recent study integrated these and other features into a network model of perivascular glymphatic exchange seeking to define the anatomical and physical features most likely to regulate perivascular transport and waste clearance [141].

A second strength of computational modeling is the ability to integrate data and processes across different biologically relevant length- and timescales. Considering length-scales, the brain extracellular space is measured in tens of nanometers, perivascular spaces range up to hundreds of microns in diameter, blood vessels span from a few microns to a few millimeters in diameter, and the cerebrovascular network encompasses hundreds of kilometers of overall vessel length. For brain waste crossing the BBB, clearance occurs over the characteristic length separating neighboring capillary vessels, typically a few tens of micrometers [142], while clearance of non-BBB-transported solutes must occur in the human brain across the centimeters of distance separating the internal CSF compartments of the ventricles and the external CSF compartments of the cisternae and subarachnoid spaces. Yet it is typically only possible to approach these processes experimentally at a single length- or timescale. EM provides the most refined anatomical view of brain tissue, yet segmentation and annotation of a single 1 mm × 1 mm × 1 mm volume by this method remains an effort of Herculean proportions. MRI provides a view into anatomy, water, and solute transport across the entire murine or human brain, yet achievable resolution frequently does not exceed 1 mm in any dimension. Computational models provide the opportunity to elaborate fine-grain anatomical detail and physical measurements at much larger scales than is achievable experimentally or permits the reduction of such details into lumped features that reflect the fine-grain experimental reality, but in simplified form to enable scaling to anatomical and temporal scales relevant to the human disease.

As noted by Bohr and colleagues, computational modeling studies have proven the most useful towards understanding glymphatic function when they are built upon an explicitly stated hypothesis, are transparent in reporting the assumptions and range of conditions for which they are applicable, can explain existing observational and experimental data, and permit the generation of subsequent falsifiable hypotheses that can be tested experimentally. For example, a recent study [71] used fluid dynamic simulations to evaluate the potential mechanisms contributing to observed perivascular fluid movements at the brain surface captured by dynamic particle-tracking studies [66]. These simulations suggested that while the oscillatory motion of particles within the perivascular spaces was attributable to peristaltic motion of the arterial wall, the net flux of particles within these spaces were more likely attributable to a standing, low-magnitude (1–2 mmHg) pressure gradient. This study, anchored by experimental data, elaborated on existing models of perivascular fluid and solute transport (e.g. [58]), and provided a clear testable hypothesis, namely that a low-magnitude pressure gradient is present between sources and sinks for perivascular fluid transport, and that perivascular transport would be sensitive to experimental manipulations of this gradient that could validate or invalidate this new piece of the field’s working model. In a second example, the above network model has been used to explore the anatomical and physiological parameters that could accomplish rapid fluid exchange through brain tissue, exhibit sleep–wake differences in function, and be driven by low-magnitude pressure gradients [141]. A sensitivity analysis based on this model suggests that parenchymal fluid transport can be highly sensitive to the size of the gaps between astrocyte endfeet, which separate low-resistance perivascular pathways from the wider interstitium. These findings suggest actionable next steps that experimentalists should take to evaluate whether these gaps are regulated to modulate solute transport across physiological conditions.

Defining the role that changes in the glymphatic system play in the development of CAA is a subject well poised to leverage computational modeling. Vascular Aβ deposition is a mesoscale phenomenon taking place at the level of individual or small populations of cerebral blood vessels. Yet this process is likely to be strongly defined by microscopic changes in the brain extracellular space and the vascular mural wall, and by macroscopic, non-local transport properties of the cerebrovascular network [143, 144] and CSF flow dynamics. The most reliable anatomical data is available from EM studies carried out under tightly controlled conditions in the rodent brain and key solute transport insights are most easily accessed by in vivo dynamic imaging approaches such as multiphoton microscopy and contrast-enhanced MRI. Yet clinically, CAA occurs in the much larger, much more complex human brain over a timescale of decades. Thus, the potential of computational modeling to integrate data from a diverse array of studies on perivascular transport and brain waste clearance, to bridge the multiple spatial scales over which these processes take place, and to link findings and mechanistic insights from the rodent brain to the wide anatomical scale of the human brain, may be key in the context of CAA. In turn, identifying which modeling assumptions are best suited to explain the transition to CAA may help understanding some of the fundamental aspects of waste clearance in the brain, providing insights on the current controversies about the glymphatic system.

## Discussion

### Alternative interpretations

As reviewed in this paper, several lines of evidence primarily from human neuropathological observations and measurements in rodent models, point towards glymphatic dysfunction being an important factor in the disease process of CAA. However, it is important to consider potential alternative interpretations regarding the observed vascular Aβ deposition patterns that do not necessarily support the theory that CAA is a result of or accompanied by impaired perivascular clearance.

Firstly, in experiments where labeled Aβ is used as a tracer, like in many rodent studies (and studies in patients using PET), it should be considered that the enhancement of cells or perivascular compartments may not necessarily be because they are implicated in perivascular brain clearance, but because they preferentially bind the tracer. For instance, the observed enhancement of arteries in rodent studies where Aβ is used as tracer may not necessarily be because arteries are the dominant clearance route. A plausible alternative explanation may be that arteries have certain features, like vascular smooth muscle cells, which possess components that potentially sequester or hinder the clearance of Aβ.

Secondly, it is well known that Aβ clearance is dependent on several other mechanisms besides glymphatic clearance, such as enzymatic degradation and transport across the BBB. It has been shown that Aβ accumulation is associated with loss of low-density lipoprotein receptors at the BBB, further mediating Aβ accumulation in the brain [145]. Studies in mice have used ^14^C-inulin, which is not cleared across the BBB, and have observed similar rates compared to perivascular clearance [69]. It remains incompletely understood whether clearance ratios across the BBB versus via the glymphatic system change over the course of the disease or with increasing CAA severity. Moreover, inter-individual differences of this ratio may result in different risk profiles for developing CAA.

Thirdly, Aβ can be co-deposited with other proteins. Recent work showed that Medin, a protein fragment of MFG-E8, deposits in the vasculature of humans over 50 years of age [146]. Medin co-localizes with Aβ deposits in the human brain and in APP transgenic mice and directly promotes its aggregation. Moreover, mice that are deficient for Medin showed a 50% reduction in vascular Aβ deposition. Earlier work from the same group showed that Medin aggregation also causes cerebrovascular dysfunction in aging mice [147]. Other proteins that tend to ‘stick’ to Aβ are plasma proteins, such as fibrin (ogen) and IgG. Neuropathological observations in human brain tissue have indeed suggested that CAA co-localizes with these plasma proteins in the walls of arterioles [148]. Additional work using in vitro assays has demonstrated the high affinity of Aβ to interact with fibrinogen and revealed that mutated Aβ, including Dutch-type CAA, results in an up to a 50-fold stronger binding affinity [149]. As such, the accumulation of Aβ in the vasculature may reflect the protein’s affinity to interact with these other proteins at the level of the BBB.

Finally, if impaired perivascular Aβ clearance is indeed implicated in the pathophysiology of CAA, it remains unclear whether initial glymphatic dysfunction results in vascular Aβ accumulation or whether early Aβ deposition in the arterioles due to potentially alternative mechanisms results in impaired clearance. Moreover, the involvement of the glymphatic system likely differs depending on the severity of the observed pathology associated with different stages of the disease course.

### Vision for future research in this field

Many unresolved questions remain regarding the basic biological mechanisms of the glymphatic system and the role of perivascular Aβ clearance in the pathophysiology of CAA. Solving these outstanding knowledge gaps requires successful collaboration between pre-clinical, clinical, and computational investigators who approach these fundamental unknowns from different perspectives. Our vision for future research in this area is to leverage the expertise of a multidisciplinary group of researchers who share the desire to advance the field towards understanding the pathophysiology of CAA and to move towards developing novel targets for treatment of this common and debilitating disease. This was the reason that we started the Leducq Foundation Transatlantic Network of Excellence on Brain Clearance. Within this network we strive to develop harmonized experimental protocols, which will be shared with the wider scientific community. The hope is that the unique interdisciplinary mix of involved investigators who have often held opposing views will accelerate translational impact by jointly interpreting and reconciling experimental findings. Moreover, we will strive to adopt minimally invasive imaging methods of the glymphatic system in rodents, catalyze development of novel MRI methods to capture fluid flow in the human brain, and integrate observations across scales and modalities in comprehensive computational models.

In conclusion, CAA represents both a highly relevant neurovascular disease as well as a helpful ‘model system’ to leverage with the goal to elucidating the current controversies concerning the glymphatic system. Insights derived from vascular Aβ accumulation patterns and CAA-related pathologies in the rodent and human brain, as well as real-time fluid flow dynamics in experimental settings have provided important clues, but the current information available on CAA is not sufficient yet, to rule out other potential explanations. Further experiments in both humans and rodents are desperately needed to fill these crucial gaps in our knowledge. Moreover, novel insights gained on the relationship between glymphatic failure and accumulation of Aβ in the vasculature will be highly relevant to understanding mechanisms of ARIA formation in the context of anti-amyloid immunotherapy, a topic of great clinical relevance.

## Data Availability

N/a.
